# Changes in retro-odontoid mass after upper cervical spine surgery

**DOI:** 10.1038/s41598-022-24436-7

**Published:** 2022-11-21

**Authors:** Jae-Won Shin, Kyung-Soo Suk, Hak-Sun Kim, Jae-Ho Yang, Hwan-Mo Lee, Sung-Hwan Moon, Byung-Ho Lee, Jin-Oh Park, Sang-Jun Park, Sub-ri Park, Sun-kyu Kim, Jane F. Garcia

**Affiliations:** 1 Department of Orthopedic Surgery, National Health Insurance Service Ilsan Hospital, 100 Ilsan-ro, Goyang, 10444 Republic of Korea; 2 Department of Orthopedic Surgery, Yonsei University College of Medicine, 211 Eonju-ro, Gangnam-gu, Seoul, 06273 Republic of Korea

**Keywords:** Diseases, Medical research

## Abstract

A non-neoplastic mass posterior to the dens is termed a retro-odontoid mass (R-OM). This retrospective study evaluated radiographic and clinical outcomes and R-OM changes after upper cervical spine surgery. This study included 69 patients who underwent upper cervical spine surgery, including atlantoaxial fusion, occipitocervical fusion, or decompression. All patients underwent preoperative magnetic resonance imaging (MRI). Six-month follow-up MRI examinations were performed in 30 patients who had preoperative R-OMs. Radiographic outcomes of the anterior and posterior atlantodental intervals were measured using X-rays and computed tomography. The R-OM and space available for the cord (SAC) were measured using MRI. Clinical outcomes were evaluated using neck and arm pain visual analog scales, the Japanese Orthopedic Association score, the neck disability index, and the patient-reported subjective improvement rate. The anterior atlantodental interval decreased, while the posterior atlantodental interval and SAC increased postoperatively. Among the clinical outcomes, the neck and arm pain and the neck disability index decreased postoperatively, while the Japanese Orthopedic Association score increased. All clinical and radiographic outcomes improved postoperatively. The R-OM either decreased in size or disappeared after fusion surgery in all cases, except in one patient who underwent decompression surgery. In conclusion, stabilization through fusion surgery is essential for treating R-OM.

## Introduction

Upper cervical spine instability in the occipitocervical (OC) or atlantoaxial area can occur following various disorders^1–4^. Without timely diagnosis and surgical treatment, severe neurologic morbidity or mortality may occur^5–7^. The complexity of surgical stabilization techniques is attributed to the peculiar anatomy of this region. Earlier techniques were often followed by increased complications and failure rates^8^, while modern fixation materials and constructs have resulted in better outcomes. Currently, screw-based constructs provide rigid, short-segment stabilization—which is crucial in achieving successful fusion—thereby preventing aggravation of neurologic morbidity and myelopathy^9^.

A non-neoplastic soft tissue mass posterior to the dens is termed a retrodental or retro-odontoid mass (R-OM); in other studies, it was also referred to as a retro-odontoid or periodontoid pseudotumor^5,10,11^. R-OMs are often observed in patients with rheumatoid arthritis, congenital anomalies, os odontoideum, degenerative conditions, or trauma. They develop because of synovial inflammation-associated atlantoaxial instability; however, some controversy exists, as some studies have stated that R-OMs can develop without the presence of inflammatory disease sequelae^12–15^.

Few comparative studies between fusion and nonfusion procedures have focused on the regression of R-OMs, and a limited number of case reports have documented the fate of R-OMs after surgical treatment. For the treatment of R-OMs, some articles have suggested surgical excision through the transoral approach or C1 laminectomy without stabilization; other studies have suggested that fusion surgery is essential^16–20^.

This study aimed to investigate the radiographic and clinical outcomes of upper cervical spine surgery and analyze how R-OMs change after decompression and fusion surgery.

## Methods

### Study design and population

The Institutional Review Board of the College of Medicine, Yonsei University, approved this single-center retrospective review (approval no.: 3-2020-0243) and waived the requirement of obtaining informed consent owing to the retrospective nature of this study. This study was performed in accordance with relevant guidelines/regulations and the Declaration of Helsinki.

The medical records of 69 patients who underwent upper cervical spine surgery due to myelopathy between August 2014 and February 2021 were reviewed; patients who underwent upper cervical spine surgery due to acute fracture or trauma were excluded, and availability of postoperative magnetic resonance (MR) images was ensured in each patient before study inclusion.

The inclusion criterion was cervical myelopathy due to upper cervical lesions; most cases had C1-2 instability or spinal cord compression at C1-2. Acute fracture or trauma cases were excluded. The diagnoses of the patients were as follows: os odontoideum (n = 10), non-union of odontoid process or neglected transverse atlantal ligament injury (n = 11), occipitoatlantal assimilation (n = 20), rheumatoid arthritis (n = 11), and degenerative arthritis (n = 17). Upper cervical spine surgery included C1-2 fusion (n = 45), OC fusion (n = 22), or decompression only (n = 2). The average postoperative follow-up duration was 25.49 ± 14.22 (6–54) months.

### Treatments and data collection

Atlantoaxial (C1-2) fusion was performed in 45 patients. C1 and C2 screws (3.5 or 4.0 mm in diameter; Synthes GmbH, Eimattstrasse 3, 4436, Oberdorf, Switzerland) were inserted under fluoroscopic guidance; C1 screws were fixed into the lateral mass or posterior arch of C1 while pedicle, pars, or intralaminar screws were inserted for C2 screw fixation. The screws were connected by a titanium rod (3.5 mm in diameter; Synthes GmbH)^21^. Decompression was performed in three out of 45 patients (C1 laminectomy, n = 2; C1 laminoplasty, n = 1).

**Figure 1:**
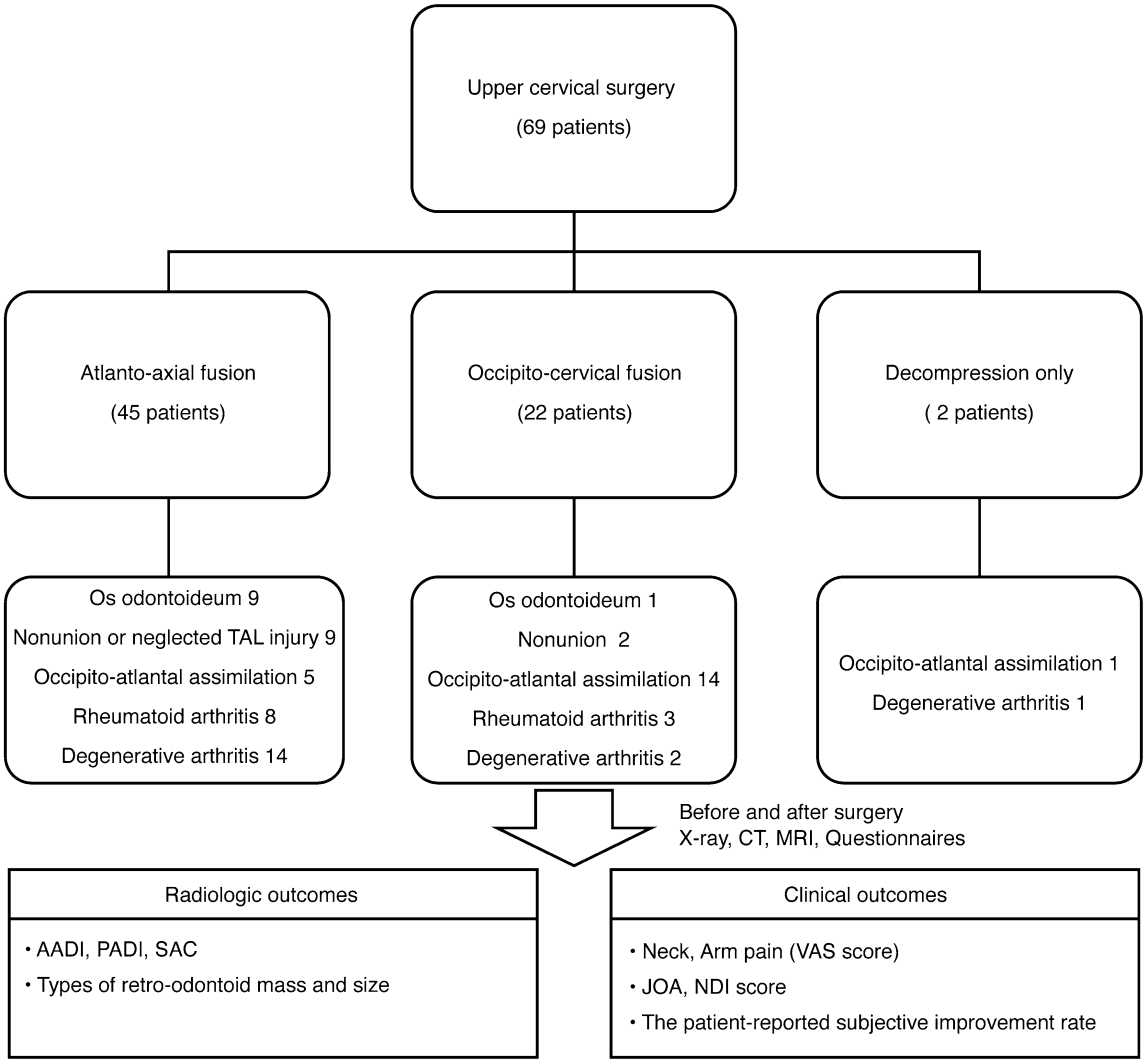
Flowchart of study participation. Sixty-nine patients presented for surgical treatment. *CT* computed tomography, *MRI* magnetic resonance imaging, *AADI* anterior atlantodental interval, *PADI* posterior atlantodental interval, *SAC* space available for the spinal cord, *JOA* Japanese Orthopedic Association Score, *NDI* neck disability index.

OC fusion surgery—which involves fusing the skull to the cervical spine—was performed in 22 patients. The occipital plate (Synthes GmbH) was contoured to the surface of the occipital bone, and bicortical screws (4.5 mm in diameter; Synthes GmbH) secured the plate to the occiput. C2 screws were inserted using the same technique mentioned above, and a contoured titanium rod connected the occipital plate to the C2 screws^22^. Among the 22 patients who underwent OC fusion, five underwent posterior decompression. Autoiliac bone grafting was performed in all fusion cases. Decompression alone (C1 laminectomy) was performed in two patients, neither of whom had any instability in the O-C2 region (Fig. 1)^23^.

All radiographs were reviewed, and all radiographic parameters were measured using Centricity (Enterprise Web ver. 3.0; GE Healthcare, Chicago, IL, USA). Pre- and postoperative lateral radiographs and computed tomography (CT) scans were used to measure the anterior atlantodens interval (AADI) and posterior atlantodens interval (PADI). The R-OM width and vertical length, and the space available for the spinal cord (SAC), were measured using preoperative and postoperative T2-weighted sagittal cervical MR imaging (MRI) findings. Preoperative spinal cord signal changes and atlantodental interval fluid collections were documented and measured using MRI. Atlantoaxial instability was characterized by excessive movement at the junction between the atlas (C1) and axis (C2) due to either a bony or ligament abnormality.

**Figure 2:**
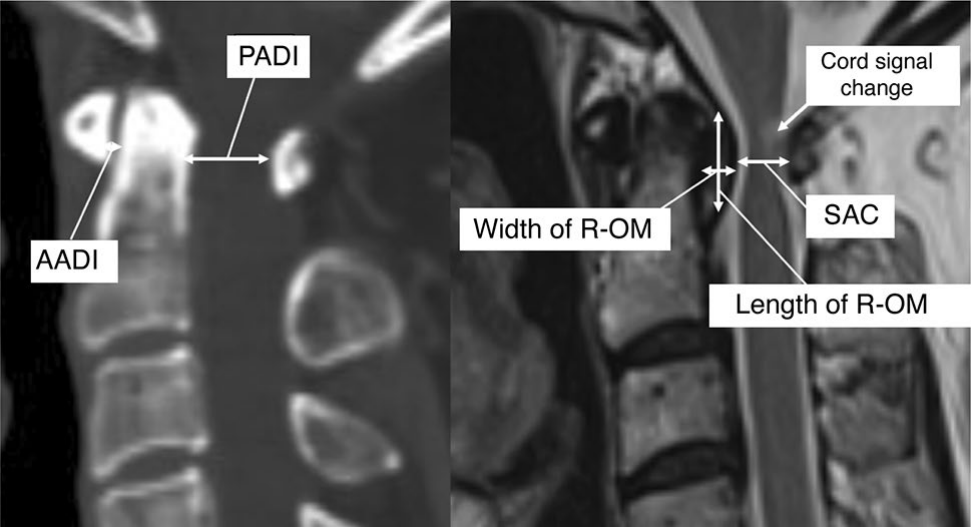
Radiographic parameters were measured on the computed tomography and T2-weighted sagittal cervical spine magnetic resonance images. *AADI* anterior atlantodental interval, *PADI* posterior atlantodental interval, *R-OM* Retro-odontoid mass, *SAC* space available for the spinal cord.

The AADI was defined as the distance between the odontoid process and the posterior border of the anterior arch of the atlas. The PADI was defined as the distance between the posterior surface of the odontoid process and the anterior surface of the posterior arch of the atlas on X-ray and CT^24^. Atlantoaxial instability was defined as the widening of the anterior atlantodental interval difference (> 3.5 mm) on the preoperative flexion–extension X-ray^25,26^. The SAC measurement was obtained using a midsagittal T2-weighted cervical MRI. The width and vertical length of the R-OM were obtained by measuring the maximum anteroposterior length and maximum vertical height of the R-OM, respectively (Fig. 2)^27–29^.

Preoperative MRI was performed in all patients, while 6-month follow-up MRI examinations were performed in 30 patients who had an R-OM noted on preoperative MRI. The diagnosis of an R-OM was based solely on MRI findings of a mass lesion posterior to the odontoid process. The size of the R-OM was documented by measuring the width and vertical length^30^. To evaluate the intra- and interobserver reliability of the radiographic parameters, two spine surgeons independently measured the radiographic parameters twice, with at least a 2-week interval between each measurement. The two independent assessors did not engage with this study and were blinded to all clinical data.

Clinical outcomes were assessed using the visual analog scale (VAS, 0–10 points) for neck and arm pain. In addition, the Japanese Orthopedic Association (JOA, 0–17 points) score and neck disability index (NDI, 0–50 points) were obtained both pre- and postoperatively. The postoperative patient-reported subjective improvement rate (IR, 0–100) was also documented^31^.

### Statistical analysis

Radiographic and clinical parameters were compared by calculating the mean and standard deviation (SD). A paired-sample t-test was used to compare patients’ radiographic and clinical parameters between the baseline and after the operation. Intra- and interobserver agreements were assessed using the intraclass correlation coefficient (ICC). The ICC with a 95% confidence interval (CI) was also calculated, comparing the mean of both trials for the two observers. Intraclass correlation coefficients < 0.40, 0.40–0.75, and 0.75–1.00 indicated poor, fair, or good, and excellent reliability, respectively^32^. All statistical analyses were conducted using SAS version 9.4 (SAS Institute, Cary, NC, USA). In all analyses, *p* values < 0.05 were considered significant.

## Results

The average age at the time of surgery was 59.12 ± 16.6 years (mean ± SD); there were 27 male and 42 female patients. Among the 69 patients who underwent upper cervical surgery, 52 exhibited a decrease in the AADI and an increase in the PADI on plain radiography and CT scans; 53 exhibited an increase in SAC on MRI findings. The mean preoperative AADIs were 4.52 ± 2.64 mm on plain radiography and 3.16 ± 2.03 mm on CT. The postoperative AADI significantly decreased to 2.67 ± 1.35 mm on plain radiography and 1.84 ± 1.24 mm on CT (*p* < 0.001 for both). The mean preoperative PADI was 13.05 ± 3.57 mm on CT; the postoperative PADI significantly increased to 15.99 ± 3.66 mm. The mean preoperative SAC was 8.38 ± 3.09 mm; the postoperative SAC significantly increased to 11.38 ± 2.92 mm (*p* < 0.001 for both) (Table 1).

**Table 1:** Comparison of radiographic results before and after surgery.

	Preoperative	Postoperative	*p* value
X-ray AADI (mm)	4.52 ± 2.64	2.67 ± 1.35	< 0.001
CT AADI (mm)	3.16 ± 2.03	1.84 ± 1.24	< 0.001
CT PADI (mm)	13.05 ± 3.57	15.99 ± 3.66	< 0.001
MRI SAC (mm)	8.38 ± 3.09	11.38 ± 2.92	< 0.001

*CT* computed tomography, *MRI* magnetic resonance imaging, *AADI* anterior atlantodental interval, *PADI* posterior atlantodental interval, *SAC*, space available for the spinal cord.

On preoperative MRI, an R-OM was found in 30 of the 69 patients (43.5%). Six-month follow-up MRI examinations were performed in these 30 patients. Ten patients (33.3%) exhibited complete regression of the R-OMs, while 19 (63.3%) had R-OMs with decreased sizes. Only one patient (3.3%) who underwent decompression surgery without fusion exhibited an increase in the size of the R-OM. All patients who underwent fusion surgery displayed a postoperative decrease in size or disappearance of the R-OM.

Cystic masses were observed in three patients; however, they completely disappeared during the 6-month follow-up period. Soft tissue masses were found in nine patients; these masses disappeared and decreased in size in two and seven patients, respectively. Additionally, a mixed type of R-OM (cyst + soft tissue mass) was found in 18 patients; these disappeared, decreased in size, and increased in size in five, 12, and one patient, respectively.

**Figure 3:**
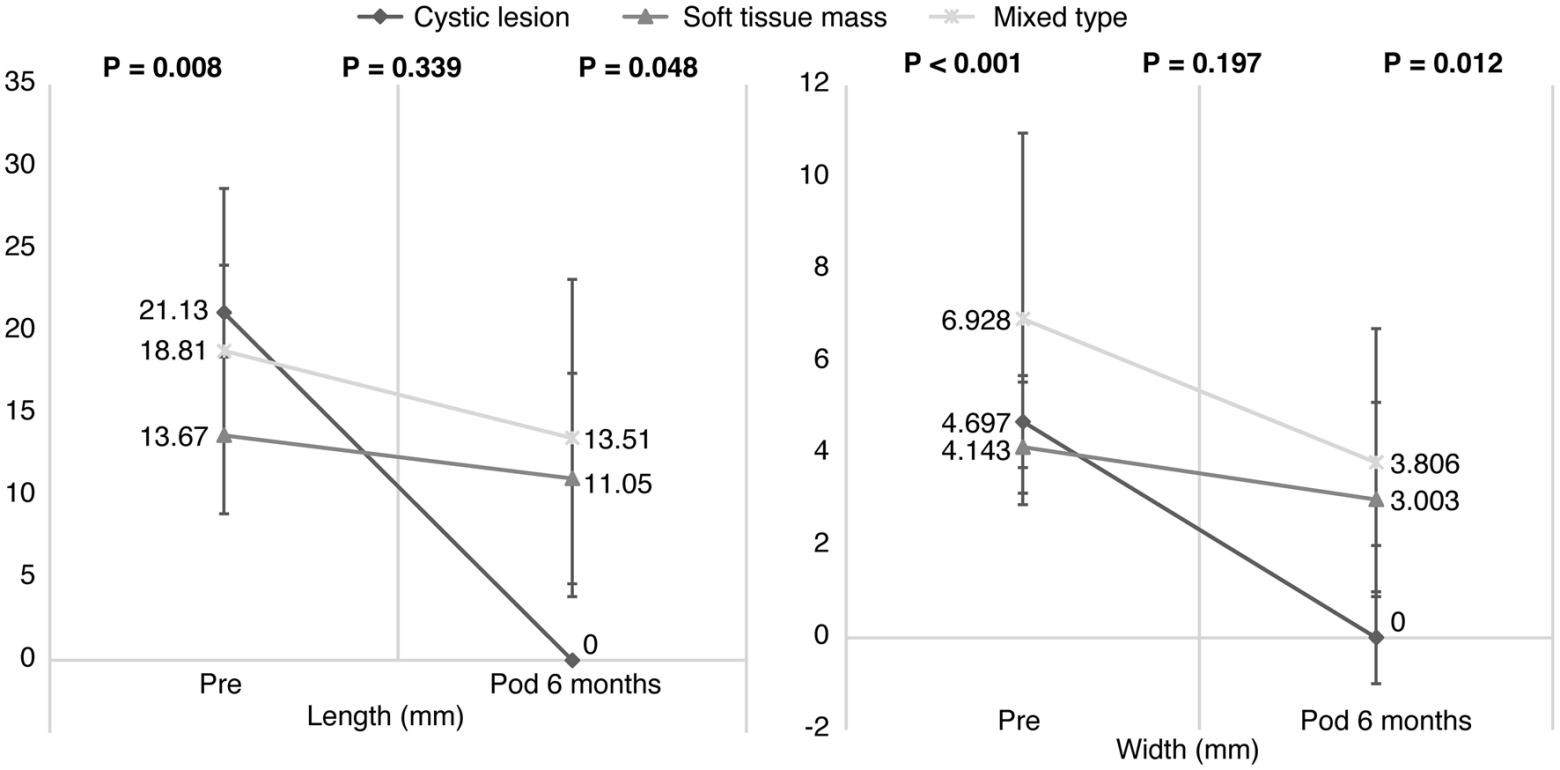
The width and length of the retro-odontoid mass were measured on T2-weighted sagittal cervical spine MRI before surgery and at 6-month follow-up examination after surgery. *Pre* Preoperative, *Pod 6 months* 6-Month follow-up after surgery, *MRI* Magnetic resonance imaging.

The mean preoperative R-OM width reduced from 5.87 ± 3.44 mm preoperatively to 3.19 ± 2.73 mm (*p* = 0.002) at the 6-month follow-up. The mean preoperative R-OM vertical length reduced from 17.50 ± 5.75 mm preoperatively to 11.40 ± 9.06 mm at the 6-month follow-up (*p* = 0.003) (Table 2). The mean width and length of the cystic masses on preoperative MRI were 4.69 ± 0.39 mm and 21.13 ± 7.56 mm; however, all disappeared at the 6-month follow-up visit (width, *p* < 0.001; length, *p* = 0.008). The mean width and length of soft tissue masses were 4.14 ± 1.41 mm and 13.67 ± 4.77 mm on preoperative MRI, and 3.01 ± 2.12 mm and 11.05 ± 6.41 mm at the 6-month follow-up (width *p* = 0.197, length *p* = 0.339), respectively. The mean width and length of mixed-type R-OMs were 6.93 ± 4.04 mm and 18.81 ± 5.21 mm on preoperative MRI, and 3.81 ± 2.91 mm and 13.51 ± 9.65 mm at the 6-month follow-up (width, *p* = 0.012; length, *p* = 0.048), respectively (Fig. 3).

**Table 2:** Comparison of R-OMs on MRI preoperatively and at the 6-month follow-up examination after surgery.

	Preoperative MRI	Six-month follow-up MRI	*p* value
R-OM (number of patients)	30	20	
Soft tissue mass	9	7	
Cystic mass	3	0	
Mixed type mass	18	13	
R-OM width (mm)	5.87 ± 3.44	3.19 ± 2.73	0.002
R-OM vertical length (mm)	17.50 ± 5.75	11.40 ± 9.06	0.003

*R-OM* retro-odontoid mass, *MRI* magnetic resonance imaging.

The clinical outcomes assessed were neck and arm pain using the VAS score, JOA score, NDI, and patient-reported subjective IR (percentage). Neck and arm pain assessed using the VAS score decreased significantly after surgery, from preoperative values of 5.05 ± 2.69 and 4.43 ± 2.56 to postoperative values of 0.45 ± 1.18 and 0.48 ± 1.27 (*p* < 0.001 for both), respectively. The JOA score increased from 11.70 ± 4.16 preoperatively to 15.39 ± 1.82 postoperatively (*p* < 0.001). The NDI decreased from a preoperative value of 21.54 ± 7.96 to a postoperative value of 13.10 ± 8.15 (*p* < 0.001). The patient-reported subjective IR was 83.89 ± 17.78%, indicating a high level of self-satisfaction after surgery (Table 3).

**Table 3:** Comparison of the clinical outcome scores before surgery and at the final follow-up after surgery.

	Preoperative	Final follow-up	*p* value
Neck pain (0–10)	5.05 ± 2.69	0.45 ± 1.18	< 0.001
Arm pain (0–10)	4.43 ± 2.56	0.48 ± 1.27	< 0.001
JOA score (0–17)	11.70 ± 4.16	15.39 ± 1.83	< 0.001
NDI (0–50)	21.54 ± 7.96	13.10 ± 8.15	< 0.001
IR (0–100)		83.89 ± 17.78	

*JOA* Japanese Orthopedic Association, *NDI* neck disability index, *IR* improvement rate.

The intra- and interobserver reliability were excellent, with the intraobserver reliability measured as 0.78–0.84 and the interobserver reliability as 0.81–0.85; however, PADI measured with radiography was found to have poor interobserver reliability (0.39); thus, the radiographic data were excluded, and PADI was measured using CT only.

No postoperative deep wound infections occurred; however, the R-OM size increased in one patient, and the clinical outcomes worsened at the 18-month follow-up visit after the decompression surgery without fusion. Consequently, posterior fusion was performed on this patient.

## Discussion

If an R-OM due to variable etiologies occurs and spinal cord compression ensues, the surgeon may be concerned regarding which surgery is appropriate. The first solution would be direct removal of the mass or decompression surgery to release the compressed spinal cord. However, this study demonstrated that the postoperative outcomes were better after upper cervical spine fusion surgery than after direct removal of the mass or posterior decompression surgery^33,34^.

**Figure 4:**
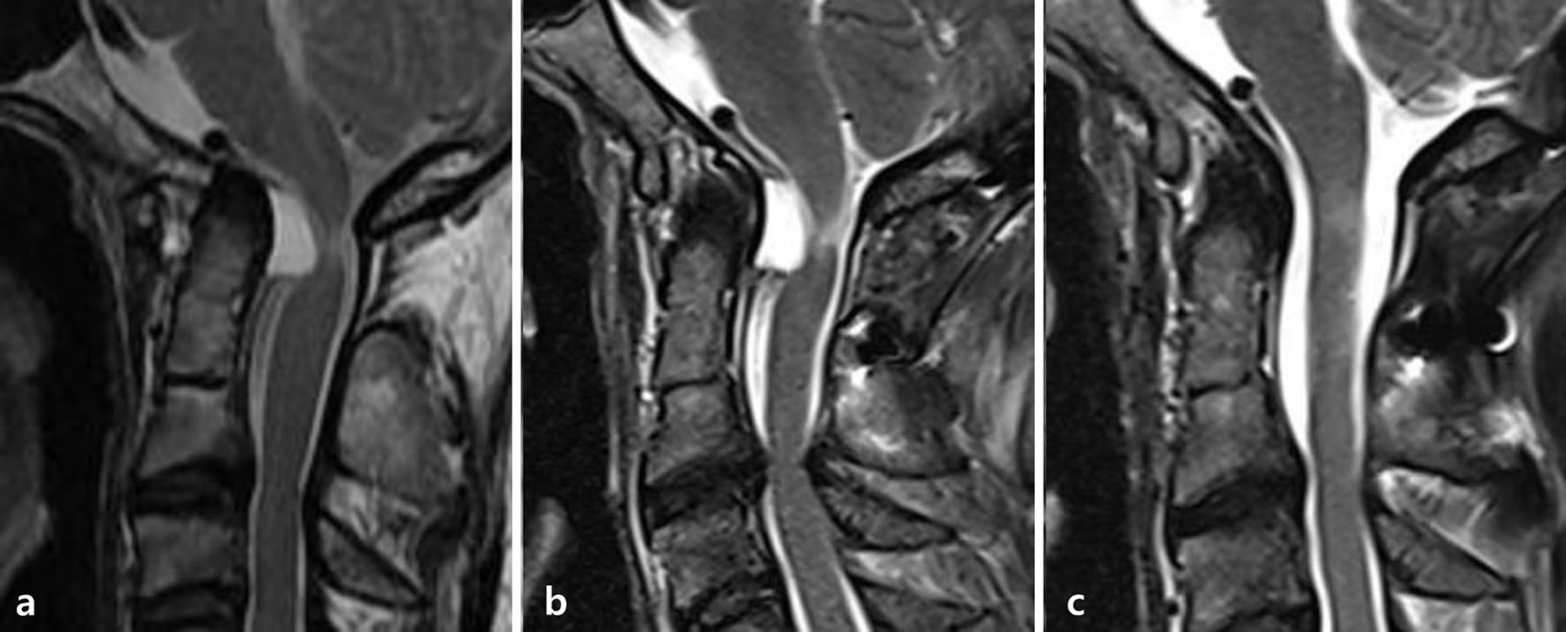
(**a**) Preoperative dynamic X-ray shows no instability at C1-2. Preoperative MRI shows R-OM with spinal cord compression. (**b**) C1-2 instability was observed at 18 months after decompression surgery. The R-OM size increased at the 18-month follow-up MRI examination. (**c**) The R-OM size decreased at 6 months after fusion surgery. *MRI* magnetic resonance imaging, *R-OM* retro-odontoid mass.

R-OM regression after decompression only has been previously reported. Kakutani et al. assessed the outcomes of C1 laminectomy for R-Oms^18^. While they observed neurologic improvements in most cases, the authors emphasized that the procedure is only recommended as a therapeutic strategy in the absence of atlantoaxial subluxation^17–19,33–37^. In the present study, two patients without preoperative upper cervical instability underwent only posterior decompression. In one patient, symptoms worsened at 18 months postoperatively, and the size of the R-OM increased. The mass size may have increased due to increasing upper cervical instability after decompression surgery or preoperative microinstability. In this patient, it was necessary to deliberate whether the fusion operation should have been considered before surgery. The preoperative T2-weighted MRI scan showed a mixed type R-OM causing spinal cord compression with associated subaxial degenerative arthritic changes (Fig. 4a). At 18 months after posterior decompression or C1 laminectomy without stabilization, the signs and symptoms of myelopathy increased. The C1–C2 instability measured using dynamic cervical radiography also increased, and the R-OM measured using MRI increased in size (Fig. 4b). Revision OC fusion was needed for stabilization, and the patient’s symptoms were relieved at the 6-month follow-up after revision OC fusion; an MRI scan confirmed almost complete resorption of the R-OM (Fig. 4c).

The clinical outcomes showed a significant postoperative improvement, which may be correlated with the postoperative size reduction of the R-OM; additionally, the upper cervical instability was reduced by posterior fusion. If the R-OM and neurologic symptoms persist after posterior fusion, additional anterior fusion may be necessary through a transoral approach. However, the results of this study suggest that the R-OM disappeared or decreased in size within 6 months in all patients who underwent posterior fusion; thus, anterior surgery may not be normally recommended^32^.

C1-2 lesions typically result from instability at the C1-2 level, causing C1-2 spinal cord compression; therefore, posterior fusion procedures should be confined to C1-2. However, in this study, OC fusion was performed in 16 patients, where some underwent occiput and C1 fusion, referred to as occipitoatlantal assimilation. In these patients, rigid stabilization was obtained through multiple fixations on the occiput^33,34,37^.

**Figure 5:**
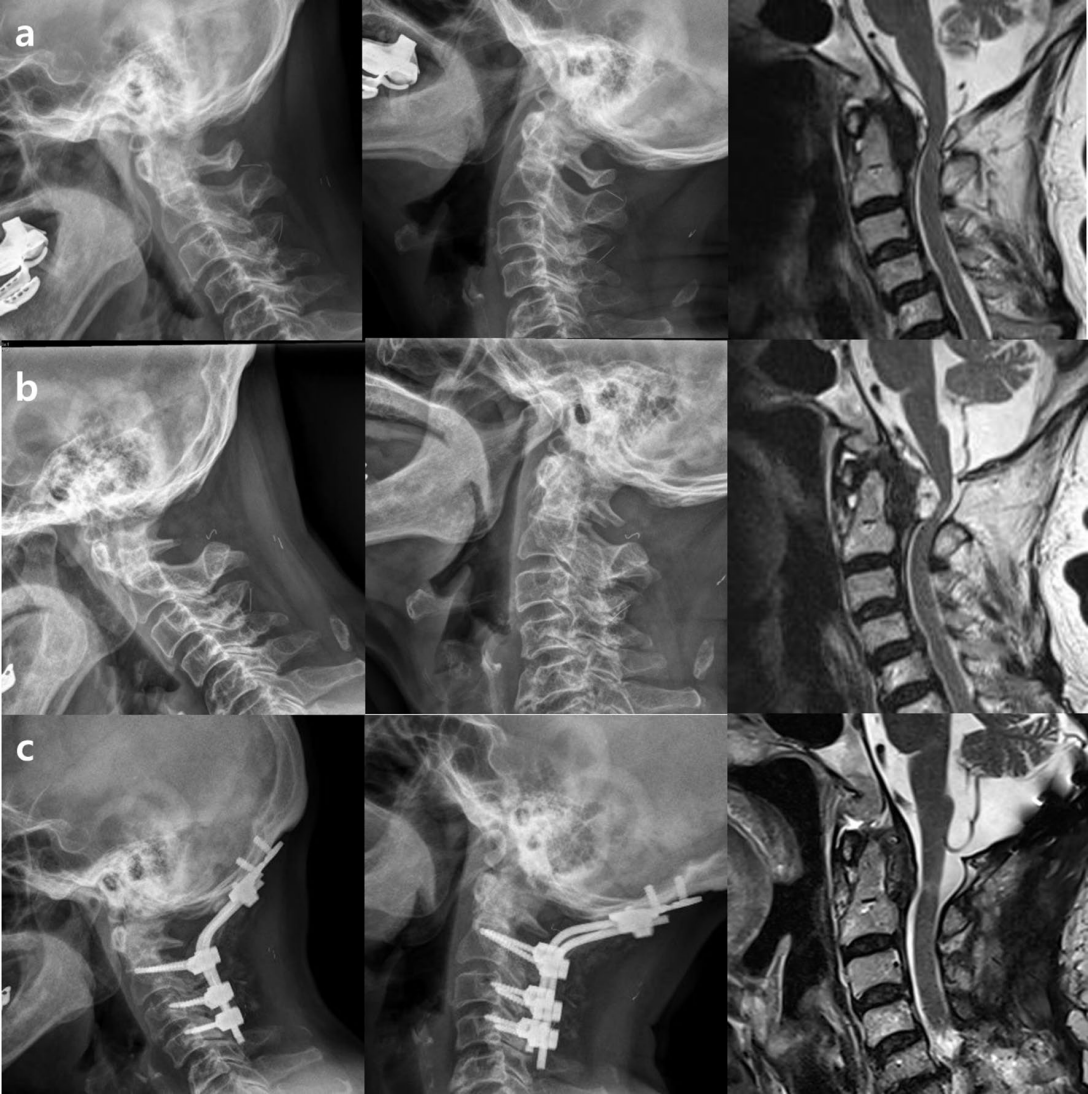
(**a**) Preoperative MRI revealing a cystic retro-odontoid mass compressing the spinal cord. (**b**) Immediate postoperative MRI shows a cystic retro-odontoid mass. The degree of spinal cord compression slightly decreased. (**c**) The cystic mass completely disappeared at the 6-month follow-up visit. *MRI* magnetic resonance imaging.

R-OMs can be classified as cystic, soft tissue, or mixed-type (cyst + soft tissue). Cystic masses disappeared from all patients at 6 months postoperatively. The average size of soft tissue masses decreased at 6 months postoperatively; however, there was no statistically significant difference. Mixed-type masses tended to completely resolve when they had a greater cystic component. R-OMs may arise from different ligaments or parts of ligaments that make up the atlantoaxial joint^26^. The mass’s most common area of occurrence was the band of the cruciate ligament. As in any inflammatory process, atlantoaxial instability or arthritis causes increased reactive fluid formation; this can cause the formation of an R-OM and atlantodental interval fluid collection (Fig. 5)^35–37^.

Two limitations were noted in the current study. First, the sample size of the surgical decompression-only group was much smaller than that of the fusion group; therefore, it cannot be generalized that the size of the R-OM increases when only decompression is performed. Second, various surgical procedures, including cervical laminoplasty and multilevel fusion, were performed in the study^33^.

In summary, patients’ clinical and radiographic outcomes for all parameters were improved after upper cervical surgery. In addition, while the R-OM either decreased in size or disappeared in all cases after the fusion surgery, it increased in size in one patient who underwent decompression surgery without fusion. In conclusion, structure stabilization through fusion surgery is essential for treating retro-odontoid masses.

## Data Availability

The datasets generated during and/or analyzed during the current study are available from the corresponding author upon reasonable request.
